# Access to the women’s Offender Personality Disorder Pathway—making criteria more gender-responsive

**DOI:** 10.3389/fpsyt.2026.1814557

**Published:** 2026-07-23

**Authors:** Aisling O’Meara, Laura d’Cruz, Ruth Horry, Carine Lewis, Jason Davies

**Affiliations:** 1 Offender Personality Disorder (OPD) Pathway Programme Team, His Majesty’s Prison and Probation Service, Ministry of Justice, London, United Kingdom; 2 School of Psychology, Faculty of Medicine, Health and Life Science, Swansea University, Swansea, United Kingdom; 3 Mental Health and Learning Disability Service Group, Swansea Bay University Health Board, Swansea, United Kingdom

**Keywords:** diagnosis and classification, forensic mental health, personality disorder, programme policy, service development

## Abstract

**Introduction:**

The Offender Personality Disorder (OPD) Pathway for England and Wales provides specialist services to men and women with complex mental health needs, which are linked to serious and harmful offending. However, criteria for accessing such services have relied on practitioner judgement and screening items developed for use with men. Within the context of a policy and practice task and finish group, this study examined existing processes and an array of routinely monitored items with the aim of developing a gender-responsive identification tool for women’s entry to the OPD Pathway that would withstand the test of time.

**Method:**

A two-part exploratory study of cross-sectional secondary data drawn from a Ministry of Justice database of all women on licence or in custody at the time of access (June 2019) was conducted. Binary logistic regression modelling of extant identification processes was carried out. Proposed alternative items indicating key characteristics that may more appropriately represent the psychological needs profiles of women on the OPD Pathway were modelled and consulted on.

**Results:**

A tool consisting of 16 items that demonstrated good predictive utility in screening outcome was established. This practical and automatable tool was rolled out for use across probation and prison services.

**Conclusion:**

This revised screening tool appeared more gender-sensitive than previous approaches to identifying women for OPD involvement while approximately maintaining the female OPD caseload figures.

## Introduction

Complex needs and mental health difficulties are highly prevalent in forensic populations (1–3) and many programmes have been developed to address various aspects of these difficulties (4–6). Within this context, the Offender Personality Disorder (OPD) Pathway was established to provide specialist resources for individuals assessed as high risk of harm who present with complex needs indicative of personality difficulties and to the staff who work with them (7, 8). These difficulties are usually associated with deprived, abusive, neglectful, or difficult childhoods (9) and can present as emotional instability or disruptive and risky behaviours that are challenging to manage within services. Since the OPD Pathway’s inception, identifying individuals appropriate for OPD services has relied upon a two-part tool on which an individual must meet set thresholds for (1) *eligibility* (determined by risk) and (2) *suitability* (determined by clinical needs indicative of a personality disorder). While men and women were identified using different eligibility criteria (due to differences in patterns of risk and offending), the same indicators of suitability for likely personality disorder were used for both groups. Over the past 10+ years, the eligibility criteria for men have remained largely consistent whilst these have evolved for women several times, including changes to offence criteria, measures of risk, and case management status. Most notably, an organisational split within the probation service in 2014 resulted in low- and medium-risk individuals being managed by private sector Community Rehabilitation Companies (CRC) whilst high- and very-high-risk individuals were managed by the public sector National Probation Service (NPS) (10). Consequently, in 2017, the eligibility criteria (risk threshold) for women were simply set as “being managed by NPS”. Reunification of probation services in 2021 resulted in eligibility criteria for women changing to a threshold of high or very high risk of serious harm or likely to need Multi-Agency Public Protection Arrangements (MAPPA) management^1^.

The initial suitability portion of the tool designed to detect likelihood of “personality disorder” was developed by Craissati and Sindall (11) and utilised 10 items from the Offender Assessment System (OASys) (12). OASys is an electronic risk assessment and management system detailing offence and sentence information, lifestyle factors, family/relationship history, socio-economic information, and attitudes (13). These suitability items were mapped to descriptors of male violent and sexual offenders meeting the earlier Dangerous and Severe Personality Disorder (DSPD) programme criteria (14), with a threshold score of seven items used to indicate likely “antisocial personality disorder”. The content of these antisocial items related to impulsiveness and aggression, predatory and exploitative behaviour, and violence and recklessness. For the OPD criteria, which sought to identify a broader range of likely personality disorder than antisocial alone, four additional indicators of “personality disturbance” were identified (15). These additional indicators (childhood difficulties, mental health difficulties, self-harm or suicide attempts, and challenging behaviour) were intended to account for characteristics of other potential personality disorders such as borderline and narcissistic presentations. In designing this tool, these authors stated the importance of incorporating such developmental variables to help identify adult personality disturbance, which they indicated as relating to sexual, physical, or emotional abuse; childhood behavioural disturbance; self-harm; and previous contact with psychiatric services (16). Instructions to probation practitioners carrying out OPD screening noted that the presence of any two of these indicators could be used where the 7/10 threshold was not met (17, 18).

While the 10 core items were directly linked to specific items contained within OASys, evidence for the presence of the additional indicators could be drawn from multiple sources of information, making the latter easier to populate and allowing two items to wield as much influence as seven. Where neither risk nor complexity criteria were met but an OPD clinician deemed it appropriate to admit an individual to the Pathway, an option to clinically override the screening outcome was also available.

Although the designers of OPD identification criteria sought to include broader presentations of “personality disturbance” than that found in the previous DSPD cohort, no specific considerations were given to women. This group, whilst proportionally very small in comparison to their male counterparts, warrants specific attention and bespoke identification criteria to ensure appropriate services and interventions. For example, official figures indicate that female prisoners are twice as likely as male prisoners to seek help for mental health problems (19) whilst the rate of self-harm amongst female prisoners is almost five times that of male prisoners (20). Studies also suggest that self-harm may be the best predictor of juvenile female violent offending, with female offenders who engage in violence shown to be significantly more likely to lose control of their temper than were non-violent female offenders (21, 22). Women who have committed violent offences have also been found to have had higher rates of suicidality and a higher likelihood of reaching clinical levels of histrionic and borderline personality disorders on the MCMI than male violent offenders (23). In exploring risk of violent reoffending amongst women, Putkonen et al. (24) found that 81% of those who violently reoffended had diagnoses of personality disorder. Relatedly, Goldenson et al. (25) demonstrated that female domestic violence offenders reported less attachment security, greater trauma experience, and a higher degree of personality pathology than a comparison group of non-offending, clinically engaged women. Specifically, the domestic violent group scored significantly higher on the Borderline, Dependent, and Antisocial subscales of the MCMI than the non-violent clinical comparison group. As regards personality presentations, Hornsveld et al. (26) explored differences between male and female violent offenders and noted significantly higher scores on neuroticism and trait anger and significantly lower scores on hostility for the female group, although small effect sizes were reported. Other relevant studies have explored the intersectionality of personality disfunction (in the form of psychopathy and personality disorder) and childhood difficulties in female prisoners (27). These authors reported that histories of suicide attempts were significantly positively associated with Factor 2 of the Psychopathy Checklist (e.g., antisocial/aggressive) and negatively associated with Factor 1 (e.g., shallow affect, superficial charm, and lack of empathy), a finding that replicated previous research with men. Looking more specifically at potential risk factors in women convicted of serious violent offences compared to non-violent women, Coleman et al. (28) found that seriously violent women were significantly more likely to have had previous violent convictions whilst non-violent female offenders were significantly more likely to have had previous convictions for acquisitive offences. Finally, whilst there is limited research in relation to women who commit sexual offences, Strickland (29) reported significant differences between female sex offenders compared with female non-sexual offenders in the amount of childhood trauma and severity of sexual abuse history experienced. The rate of sexual victimisation amongst female sexual offenders has also been found to distinguish them from female offenders who commit violent offences and men who commit violent or sexual offences (30). Additionally, female sexual offenders have been found to have significantly less alcohol and substance abuse history than male violent and sexual offenders.

Given the above research, the male-focussed content of the established OPD suitability criteria (being based on the characteristics of men within the DSPD programme), and different personality presentations being described for men and women within the criminal justice system (CJS) more broadly (7), a multi-disciplinary task group was created to establish a gender-responsive identification process for OPD services that would withstand further organisational change. The present study was commissioned as part of a PhD project (31) to determine (1) the performance of the current set of 10 OASys items and four additional indicators in identifying women for entry to OPD services and (2) how this process might be improved.

## Materials and methods

This work was carried out in two stages and utilised mixed methods. Stage 1 involved analysis of the extant suitability criteria to evaluate the applicability of this tool to a subset of women using comparative statistics and regression modelling. Stage 2 consisted of scoping alternative items for identifying women as suitable for OPD involvement and analysing these for the full female dataset using binary logistic regression modelling to evaluate the predictive power of newly proposed items. Approval was obtained from the HMPPS National Research Committee (Ref: 2017-342). As this work consisted of secondary data analysis on systems-based data, no ethical approval was required to conduct this study. Consent was not required as this study utilised systems-based data that were processed under UK GDPR Article 6.1 Lawful Basis for processing: (e) processing is necessary for the performance of a task carried out in the public interest or in the exercise of official authority vested in the controller.

### Transparency

This study’s design and its analysis were not preregistered publicly; however, the details of intended analyses were reviewed and approved by the HMPPS National Research Committee prior to commencement of the study. The data that support the findings of this study are not available to readers as analyses were conducted on secondary data accessed through Ministry of Justice recording systems, which are not available for public information. Analytical outputs and processes utilised throughout the analysis are available as annexes attached to the doctoral thesis that encompasses this study (31). Data were analysed using R version 3.6.1 (CRAN, n.d.) and SPPS version 22 (IBM, n.d.).

### Participants

A dataset of 16,307 women was available, representing 8.6% of the total prison and probation population across England and Wales as at 30 June 2019. Of these, 3,761 were managed by the NPS and 12,546 were managed by CRC. The number of women on the OPD caseload at the time data were accessed was 1,869, accounting for 50% of NPS women (11.5% of all women subject to prison or probation).

### Data available for analysis

Variables included in this dataset were general demographics (age, ethnicity, and geographic region), all OASys tick-box items, offence and sentence information, and risk scores including violence predictors, general reoffending predictors, serious reconviction, and clinical risk assessments. Tick-box items within OASys are scored either 0/1 or 0/1/2, and two additional items at the end of each section ask whether the section topic is (1) linked to risk of serious harm, risks to the individual, and other risks, and (2) linked to offending behaviour. Additional variables, drawn from the OPD Pathway database, indicated the method by which women on the OPD caseload had been identified as suitable for OPD services (i.e., through one of the two thresholds—7/10 core or 2/4 additional indicators—or through clinical override). As the four additional indicator items did not relate directly to specific single OASys items, a set of proxy indicators (each comprising several OASys items, any of which counted as item presence) was devised by the MoJ Data Sciences Hub for the purpose of this study and included in the dataset.

### Stage 1 method

#### Design

A cross-sectional design was employed to establish differences between OPD and non-OPD women. Following this, the OPD suitability criteria were assessed in terms of their predictive power in identifying women who had been deemed suitable for OPD services.

#### Participants

Analyses of the extant criteria were run on the NPS portion of the data (*n* = 3,761) only as all would be deemed eligible for OPD consideration based on the criteria in operation at the time. Ages ranged from 19 to 83 (*M* = 38.18, *SD* = 11.51) and ethnicity proportions are presented in **Table 1**. Additional demographic and offence-related characteristics are available from the thesis in which this study was conducted (31).

**Table 1:** Ethnicity proportions for women on the OPD caseload compared with wider probation caseload.

Ethnicity	*N*	% of NPS	On OPD database (% of OPD)	Rate of screening in by ethnicity
White	3,099	82.4%	1,600 (85.6%)	52.6%
Mixed	177	4.7%	104 (5.6%)	58.8%
Black	286	7.6%	96 (5.1%)	33.6%
Asian	121	3.2%	48 (2.6%)	39.7%
Not known	48	1.3%	11 (0.6%)	22.9%
Other	30	0.8%	9 (0.5%)	30%
*Total*	*3,761*	*100*	*1,868*	*49.7%*

#### Analysis

Independent samples *t*-tests compared total scores on the 10-item screen for women on the OPD Pathway with those not on the OPD caseload. Chi-square analyses were used to explore the relationship between the 10 OASys items and presence on the OPD caseload, with odds ratios provided to indicate effect sizes of item function. To explore the predictive utility of each item and the four additional indicators, logistic regression analyses were carried out and the resultant model’s predictive strength was calculated.

### Stage 1 results

#### Reliability

A Cronbach’s alpha value of 0.626 was achieved for these 10 items, with a mean scale score of 4.73 (*SD* = 2.11) and a skewness of −0.133, indicating a close-to-normal distribution of scores. The suggested removal of one item (“offence involved excessive violence or sadism”) would have improved reliability to *α* = 0.650, indicating a moderate level of internal consistency. As this stage of the analysis sought to establish scale characteristics, no items were removed during this phase.

#### Group differences

**Figure 1:**
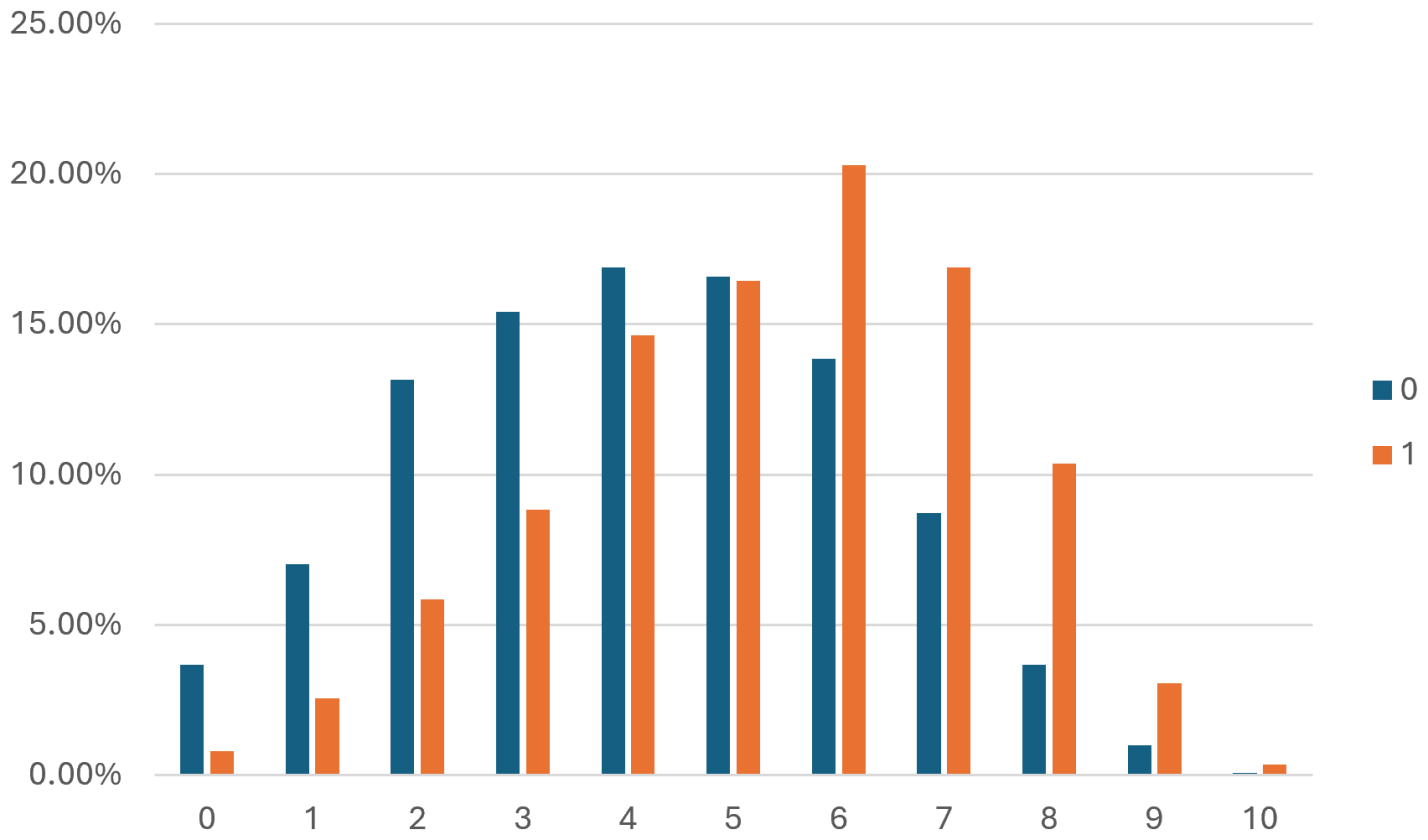
Percentages of item scores for screened in vs screened out NPS women.

Demographic differences between OPD women and the general NPS caseload were explored, indicating comparable distributions across age, offence types, and location (prison/probation). Some differences in ethnicity were noted, as presented in **Table 1**. There was a significant mean difference of 1.21 (*t* = 18.382; *CI* = −1.343:−1.084; *p* < 0.001) between total screen scores of those on the OPD Pathway (*n* = 1,868; mean = 5.31) and those who were not (*n* = 1,893; mean = 4.10). While those screened in had significantly higher scores, both totals were below the seven-item threshold. The distribution of women in each group according to the total number of items present shows a lack of discrimination, especially for those with a total score of 5 (see **Figure 1**). A significant but weak correlation (*r* = 0.287; *p* < 0.01) was found between total score and screening status (in or out), further suggesting that the number of items identified as present had limited utility in determining screening status. Chi-square analyses indicated that all items were significantly more likely to be present for those screened in than screened out. However, with most odds ratios under 1.40 and a maximum of 1.65 achieved, very low effect sizes were evident on all comparisons, indicating the limited utility of the existing 10-item tool.

#### Role of items in determining screening outcome

The predictive function of each of the 10 core items was explored through regression analyses carried out in the RStudio environment. Simple logistic regression was first used to examine the possibility of a nested structure within the data (32) and to what extent, if any, nesting might affect the true impact of independent variables on outcomes. The benefits of incorporating random effects into regression modelling are particularly clear when working with real-world data, where assumptions around independence are easily violated. Four variables (geographic region, ethnicity, sentence type, and offence type) were considered as potentially creating non-independence in the outcome variable; thus, each of these was run through a simple regression model with OPD status (in/out) as the outcome. Significant differences were found between some levels of each of these four variables, indicating all four should be included as random intercepts in prospective regression models of OPD status (33). To further explore the role of these variables in predicting screening outcome, an intercept-only model was run and compared against each iteration of the mixed-effects model incorporating all four random intercepts. An AIC of 5,017.2 was achieved by the intercept-only model, decreasing with the addition of each random intercept to 4,713.1. This supported the inclusion of these variables as random effects in further models of screening outcome.

To explore the predictive power of the 10 items, individual dichotomous items were modelled alone first, followed by the combined 10 items as fixed effects with the four random intercepts included. Modelling was fit by maximum likelihood with Laplace approximation (34) and **Table 2** presents the output from this mixed-effects model. The reference category (Intercept) presents the log likelihood of screening in when all predictor variables are equal to 0 (i.e., not present). The estimate indicates the change in log likelihood of screening in for each consecutive item when all other items are absent, and the *z* value indicates the number of standard deviations that the estimate is from 0 (with a minimum value of 1.96 required to reach statistical significance at *p* < 0.05). The highest predictive value was achieved by item 8 (“evidence of childhood behaviour problems”), such that having this item increased the log odds of screening in by 0.74—an odds ratio of 2.1:1 for screening in if this item was present vs. absent.

**Table 2:** Output from mixed effects regression model of 10 core items on OPD status.

AIC	BIC	Log Lik.	Dev.	df.resid
4,426.2	4,519.1	−2,198.1	4,396.2	3603
Fixed effects:				
	Estimate	Std. error	*z*-value	Pr(>|*z*|)
(Intercept)	−1.48128	0.34987	−4.234	2.30e−05***
PD1 conviction under 18	0.11026	0.08476	1.301	0.193288
PD2 offence involved violence/threat of violence/coercion	0.27028	0.08888	3.041	0.002357**
PD3 offence involved excessive violence/sadism	0.41964	0.09831	4.268	1.97e−05***
PD4 fails to recognise impact of offending on victim or society	0.11394	0.15948	0.714	0.474953
PD5 overreliance on others for financial support	0.05944	0.07951	0.748	0.454731
PD6 manipulative or predatory lifestyle	0.22641	0.08405	2.694	0.007066**
PD7 reckless risk-taking behaviour	−0.03682	0.10842	−0.340	0.734157
PD8 evidence of childhood behaviour problems	0.74028	0.08812	8.401	<2e−16***
PD9 impulsivity	0.40702	0.10486	3.882	0.000104***
PD10 aggressive or controlling behaviour	0.58151	0.09291	6.259	3.88e−10***

Significance codes: 0 “***”; 0.001 “**”; 0.01 “*”; 0.05 “.”; 0.1 “ ”; 1.

The proxy items developed to represent the aforementioned four additional indicators were also analysed to establish their predictive power in identifying women on the OPD caseload, and this four-item model achieved an AIC of 4,249.7, considerably better than that achieved by the 10-item model. All four proxy indicators significantly increased likelihood of screening in, with Childhood Difficulties (a combination of two items) and Mental Health problems (a combination of seven items) having particularly large estimates. Finally, a model incorporating all 14 items achieved an AIC of 4,167.9, indicating a better fit than either the 10-item or indicator-only models. However, many variables yielded low estimate values and were not statistically significant predictors of OPD status. Through an iterative process of removing non-significant items and re-running analyses, the best-fitting model comprised five OASys items (violence/coercion, extreme violence/sadism, manipulative/predatory lifestyle, childhood behaviour problems, and aggressive/controlling behaviour) and all four proxy items for the additional indicators. This model achieved an AIC of 4,162, indicating a slightly better fit given the reduced number of component parts. An analysis of variance (ANOVA), utilising χ^2^, run between the 14-item and 9-item models indicated that this was a non-significant improvement (χ^2^ = 4.068; *df* = 5; *p* = 0.540) and a train and test model indicated a predictive accuracy of 71%.

### Stage 1 summary

While analysis suggested that the most parsimonious solution was achieved with a set of nine items, this was not deemed an acceptable solution to the female OPD screening question given these included non-mandatory OASys items, overlapping content, combination items (e.g., proxies), and unimpressive odds ratios.

### Stage 2 method

#### Design

An exploratory approach was taken to identify a more efficient and useful set of identification criteria. A large pool of OASys items was selected, in collaboration with the multi-disciplinary task group, to be analysed through binary logistic regression modelling on the single outcome of OPD caseload status (in/out). Various criteria for identifying women for OPD involvement were used over the years, meaning women on the OPD caseload were not a homogeneous group. This cohort was composed of women meeting core criteria; those who scored low on the 10-item screen but demonstrated significant mental health needs through additional indicators; and women who, by professional opinion, were clinically overridden into the Pathway despite meeting no established criteria. As such, in lieu of a formal diagnosis, presence on the OPD caseload was what distinguished these women from the wider national caseload. Owing to the real-world nature of this research, a more appropriate outcome variable for identifying women who “should” be on the OPD caseload was not available. All variables under analysis were regressed onto the single outcome variable of OPD status, and the resultant set of items were evaluated for appropriateness.

#### Item identification

Items considered for inclusion consisted of 141 tick-box questions from sections 2–13 of OASys, excluding drug and alcohol factors as initial inspection of the data indicated these were unlikely to discriminate between OPD and non-OPD women. To identify items for analysis, four steps were taken. Items were first mapped against the DSM V and ICD-11 criteria for personality disorders; 22 OASys items mapped on to criteria for one or more personality disorders and were selected for inclusion. Second, items were mapped against the overarching OPD strategy and current screening tool. Four items mapped onto offence categories indicated as key focus areas in the OPD strategy; eight items were retained from the original criteria; two items were closely representative of the indicators in the original tool; and four items, previously identified as highly correlated with women’s current screening status during an initial inspection of the data, were included in the item pool. Third, a brief snowball survey asked members of the women’s prison and probation estates, with mental health and/or OPD-specific functions, to indicate factors they considered relevant in identifying women for OPD inclusion and to comment on the functionality of the current tool. From 18 responses received, three items that had not already been mapped were identified. As anticipated, risk of harm to others was ranked the most important factor to consider when determining OPD Pathway inclusion, with 10 out of 18 respondents ranking this factor in first place and an additional three respondents ranking this the second most important factor. Traumatic histories were the next most highly ranked factor followed by emotional regulation difficulties. Finally, the complete list of OASys tick-box items from sections 2–13 was presented to the task group with previously identified items highlighted and noted as to source. Members were asked to indicate whether any further items should be included in the analysis. Six members suggested additional content amounting to a further 25 items put forth for analysis. From this four-stage process, a pool of 68 items from multiple discovery sources were taken forwards for predictive modelling. Given the absence of knowledge regarding defining characteristics of the female OPD Pathway caseload, this large and diverse item pool ensured that a wide range of potentially relevant items were included for analysis.

#### Participants

Of the available full dataset of 16,307 women, 3,823 women were removed because of missing values in one or more of the 68 variables under analysis. Consequently, data from 12,484 women (NPS = 3,381; CRC = 9,103) were analysed. As this model-building process was not an attempt to discover which items were diagnostic for having been on the Pathway but rather aimed to predict which women could be on the Pathway in the future, data from both NPS and CRC women were included.

#### Analysis

Data were prepared for modelling in the RStudio environment, ensuring that formatting of response categories was consistent and comparable. The random intercepts incorporated into NPS modelling were also tested to determine whether mixed-effects modelling was again required. An intercept-only model was run first, followed by each of these variables previously identified as creating non-independence in the outcome of OPD status. Individual OASys items were then analysed through generalised linear mixed modelling (GLMM) with random intercepts factored in. Items were tested in order of appearance in the OASys tool and relevant data were logged to inform order of imputation in combined items modelling. Combinations of items were then modelled by binary logistic regression within GLMM to establish the best-fitting collection of items for OPD screening outcome. Once a suitable set of items was identified, the task group was consulted to identify pertinent points for consideration regarding item inclusion and threshold setting.

To identify which items in combination would have the best predictive value, binary logistic regression models of items with the highest estimates were run. Stepwise entry was utilised for model selection. This was an iterative process, with one item added at a time to continuously monitor the model fit of combinations of items. Items were removed from subsequent models if they failed to improve the model fit statistics, and this was determined by either the AIC increasing or the item achieving a non-significant estimate in the combined model.

### Stage 2 results

#### Item selection

An iterative approach to model-building was taken starting with the two most predictive items. Addition of items continued, with items retained if they both improved the model fit statistics (indicated by a smaller AIC) and continued to demonstrate significant predictive value (as indicated by a large estimate) in combination with all other model components. During this selection process, two items warrant specific note. The addition of item 10.1 “Difficulties coping” improved the AIC whilst presenting with a negative log likelihood value of −0.45989 alongside an intercept of -5.65946. The correlation of fixed-effects table revealed that the effects of items 10.1 and 10.2 “Current psychological problems/depression” were highly negatively correlated at *r* = −0.450, indicating that in the absence of 10.2, it was more likely that 10.1 would also be absent. The better model fit statistics achieved by including 10.1 meant both items were retained. Model building continued until no further improvements were found and several subsequently added items presented as non-significant or weakened the overall model fit statistics. This status was reached after 39 items had been tested. Review of the final model (see **Table 3**) revealed two items that no longer reached significance; however, as their removal resulted in a higher AIC, retaining these items was examined. An ANOVA between models with and without these two items indicated a non-significant difference (χ^2^ = 5.7527; *df* = 2; *p* < 0.1); thus, the full 20 items of the final model were presented to the task group for discussion.

**Table 3:** Output from final model–mixed effects regression model of the 20 best-fitting items.

AIC	BIC	Log Lik.	Dev.	df. residual
5,159.7	5,345.5	−2,554.9	5,109.7	12,459
Fixed effects:	Estimate	Std. error	*z*-value	Pr (>|z|)
(Intercept)	−5.96758	0.51656	−11.552	<2e−16***
Analysis of offence issues linked to risk of serious harm, risks to the individual, and other risks.	1.16894	0.16474	7.096	1.29e−12***
Thinking/behaviour issues linked to risk of serious harm, risks to the individual and other risks	0.59079	0.13577	4.351	1.35e−05***
Issues of emotional wellbeing linked to risk of serious harm, risks to the individual, and other risks	0.31783	0.10641	2.987	0.002819**
Experience of childhood	0.73528	0.09745	7.545	4.51e−14***
Self-harm, attempted suicide, suicidal thoughts or feelings	0.71412	0.09077	7.868	3.61e−15***
Relationship issues linked to risk of serious harm, risks to the individual, and other risks	0.14064	0.10092	1.394	0.163433
Current psychological problems/depression	0.44206	0.13060	3.385	0.000712***
Lifestyle and associates issues linked to risk of serious harm, risks to the individual, and other risks	0.28617	0.09055	3.160	0.001576**
Difficulties coping	−0.40764	0.14288	−2.853	0.004330**
*Ever been on medication for mental health problems in the past*	0.45088	0.07724	5.838	5.29e−09***
(Did offence involve…) Any violence or threat of violence/coercion	0.39246	0.08736	4.492	7.04e−06***
(Did offence involve…) Arson	1.42284	0.21146	6.729	1.71e−11***
*Evidence of childhood behavioural problems*	0.43708	0.08557	5.108	3.26e−07***
Accommodation issues linked to risk of serious harm, risks to the individual, and other risks	0.26288	0.07974	3.297	0.000978***
*Attitude towards staff*	0.30307	0.08244	3.676	0.000237***
(Evidenced motivation) Emotional state of offender	0.16298	0.08618	1.891	0.058610.
(Did offence involve…) Carrying or using a weapon	0.64637	0.08304	7.784	7.05e−15***
*Manipulative*/*predatory lifestyle*	0.29776	0.08242	3.613	0.000303***
(Did offence involve…) Excessive use of violence/sadistic violence	0.70990	0.10785	6.582	4.63e−11***
Evidence of domestic violence/partner abuse (perpetrator)	0.24605	0.08241	2.986	0.002828**

Significance codes: 0 “***”; 0.001 “**”; 0.01 “*”; 0.05 “.”; 0.1 “ ”; 1. Items in bold are those which did not reach statistical significance in the final regression model.

Because of the real-world nature and policy implications of this research, the final revision of the women’s screening tool could not be guided by statistics alone, hence the need for a mixed-methods approach and expert consultation. The task group decided to remove four non-mandatory OASys items (**Table 3**—items in italics) from the tool to avoid arbitrary differences in total score (based on the non-mandatory nature of the item rather than the presence/absence of an item). The two items that did not reach statistical significance (**Table 3**—items in bold) were considered clinically relevant to the expression of personality difficulties and retained, despite their inclusion resulting in very slightly weakened model fit statistics.

The resultant 16-item screen was found to have good internal consistency, achieving a Cronbach’s alpha of 0.840 when analysed for the full dataset of 16,307 women, a considerable improvement on the original 10-item tool. The mean scale score across all women was 6.17 (*SD* = 3.83; variance = 14.648) with a skew of 0.203, indicating a close-to-normal distribution of scores. The removal of one item (Did the offence involve - Arson) very slightly improved reliability to *α* = 0.843; however, given the purpose of this tool and the clinical relevance of personality disorder in relation to fire-setting (35, 36), no further items were removed.

#### Setting a threshold for OPD inclusion/exclusion

When determining a threshold point for OPD inclusion, it was important to consider the consequences of incorrect classification (37) and thus to minimise both false positives (which could over-stretch OPD resources) and false negatives (which could exclude those for whom inclusion would be warranted). Based on the distribution of scores across the full dataset of 16,307 women, the suitability threshold scores of were agreed with the task group for initial testing alongside eligibility criteria to indicate resultant caseload figures. As **Table 4** demonstrates, 2,688 women met risk criteria and reached a threshold of 8/16, whilst a threshold of 10/16 would identify 2,085 women for inclusion in the OPD Pathway. Compared against the existing OPD caseload status of each of the women, a threshold of 8 resulted in a sensitivity of 86% and a specificity of 93% (precision rate of 60%) whilst a 10-point threshold resulted in a sensitivity of 74% and a specificity of 95% (precision rate of 66%).

**Table 4:** Screening outcome for 16-item tool compared against OPD caseload.

Screen format		Totals ↓	Screen in by 8+ on 16-item screen		Screen in by 10+ on 16-item screen	
			Yes	No	Yes	No
	**Totals →**	**16,307**	**2,688**	**13,619**	**2,085**	**14,222**
On existing OPD caseload	Yes	**1,869**	1,603	266	1,383	486
	No	**14,438**	1,085	13,353	702	13,736

The task group discussed the implications of each threshold and associated caseload increases, and opted to maximise specificity and precision, given the rarity of the “condition” being assessed; thus, the more restrictive threshold of 10 was adopted. This was also supported by most respondents to a consultation of stakeholders and practitioners in relevant fields. An ROC curve was produced, and the resultant AUC was 0.846 (SE = 0.006 under nonparametric assumption), which was deemed adequate. Taken in conjunction with the finding that half of survey respondents thought too few women were identified by the current tool, a 66% precision rate was acceptable. This tool identified 13% of the full female caseload as appropriate for OPD involvement, and this rate was broadly replicated across White (13.3%), Black (13.3%), and Asian (14.2%) groups, although it tended to slightly over-identify Mixed race women (17.6%) and under-identify unknown/other ethnicities (10.2%).

#### Interim review

An interim review of this tool in 2024 indicated that an appropriate number of women were being identified for OPD Pathway inclusion, maintaining a 13% identification rate. The eligibility section of the tool screened out a higher proportion than did suitability criteria, with just 15% of women meeting eligibility criteria whilst 35% met the 10/16 threshold. Analyses also revealed that a lower threshold of 8/16 would have increased identification rates to almost 15% of the caseload. The introduction of automated screening and recorded use of clinical override in 2024 allowed for a small dataset covering 5 months of activity. This demonstrated a clinical override rate of 5.4% during this period.

## Discussion

This study was the first to investigate criteria for suitability for OPD services within a female offending population using robust statistical methods, which, following consultation and approval, were implemented across England and Wales in June 2021.

### Item types

There were several observable groupings of item types within the final suitability criteria presented in **Table 3** (non-italics). Six items indicated specific OASys categories as linked to risk of harm (e.g., “Thinking/behaviour issues linked to risk of serious harm, risks to the individual, and other risks”). These items may perform a bridging function in connecting relevant psychological factors to risk whilst not directly implying specific risk features themselves. It is unsurprising that relationships, lifestyle, wellbeing, and thinking styles all feature here, with much of the literature on women’s violent offending indicating that relational difficulties resulting from insecure attachment styles, experiences of childhood trauma, and neglect all interconnect to increase the risk of recidivism (38) and violence in women (22) and that this is aggravated by policies that do not address these needs appropriately within judicial services (24, 39). This item type effectively mirrors the overarching OPD criteria requiring a “clinically justifiable link between the personality disorder and offending” (40), allowing these links to be made to the broader concepts captured where specific items in these sections of the assessment may not suitably reflect such links. Previous studies have specifically indicated higher rates of suicidality, self-harm, and emotional dysregulation in women convicted of violent offences (22, 25), and these factors are borne out in the resultant screening items. Three items directly referenced self-harm and psychological difficulties, which have been found to present more frequently in female offenders with psychiatric morbidity (41).

Items relating to past and present interpersonal issues importantly reflect the bio-psycho-social framework underpinning the principles of the OPD Pathway (42), with previous studies noting how unacknowledged traumatic experiences can lead to persistent and complex mental health conditions (43), poor attachment formation, and difficulties developing positive interpersonal relating styles (44). This, in turn, can lead to behavioural effects such as violence and aggression (45), with some studies indicating that women in forensic settings with personality disorder diagnoses were more likely to have arson convictions and incidents towards self and others were of a more violent nature than women in forensic settings without a personality disorder diagnosis (36, 46), reflecting the five items relating to offence characteristics. Considering the relational approach purported by the OPD Pathway, it is notable that literature on women offenders have identified supportive relationship styles as positively impactful on women’s ability to reduce anxiety, with more punitive approaches compounding a sense of injustice and reducing motivation to engage (47). These provide indicators of potential approaches to intervention that may be particularly important when working with women.

### Real-world applicability

Strengths of this study included the thorough approach taken to identify OASys items to replace the extant suitability criteria. Utilising established knowledge around personality disorder and types of offending amongst women (46) was important in identifying relevant OASys items for initial modelling. Additionally, input from frontline and strategic professionals ensured real-world applicability of the resultant criteria and scoping potential caseload outcomes at various decision-making stages ensured processes remained aligned with study objectives.

This study was strengthened by its position as both a policy-informed and policy-informing exercise. A gender-responsive approach to identifying women for OPD intervention was lacking, and this study addressed this gap in service provision. While exploration of screening content was limited to the OASys, this can simultaneously be seen as a strength given that all individuals convicted of offences in England and Wales are subject to regular OASys assessments. As such, this limiting factor allows for all individuals to be routinely assessed for inclusion, without affecting resources. Recent research has noted the difficulties experienced by women with criminal histories in accessing appropriate psychological input to address personality-related issues in the community, noting that the identification of personality disturbance upon entry to prison may result in better treatment opportunities and access to relevant services (48). With this in mind, the integration of the OPD screening process into routine assessment is an important and useful policy development.

### Limitations

It is notable that this study did not specifically address risk assessment factors in spite of the target group being individuals with complex psychological needs who present with a high risk of harm to self or others. Risk assessment instruments have typically been developed utilising data drawn from male offenders, and there have been many debates addressing their applicability and efficacy in the female offending population. Some studies have found no differences in the accuracy of prediction between genders, others have demonstrated issues related to the nature of risk predicted or the focus of the tool used, whilst others have demonstrated mediating effects of personality or lifestyle factors on differences in accuracy across genders (49–52). In the present study, the task group set up to revise these criteria had already identified several alternative approaches to screening women that considered offence types and various risk metrics embedded into administrative systems. This element of the screening process was therefore not presented for thorough exploration or amendment in conjunction with the suitability factors, and the resultant eligibility criteria of high risk of serious harm or being MAPPA managed were determined by a previous analysis.

Another limitation was the inclusion of non-mandatory items in the modelling process, which provided unnecessary information and potentially led to the exclusion of participants from the modelling sample given that these items were deselected due to their non-mandatory format rather than their model performance. This learning will be taken forwards to the process currently underway to examine the OPD criteria for men. An unavoidable limitation was the use of current screening status as the outcome on which all items were regressed, which assumed that those on the OPD caseload at the time of analysis were correctly identified. This condition was a factor of the real-world nature of the study and a consequence of the service design and, in the absence of an objective standard of suitability, remains the most practical and feasible outcome available.

### Constraints on generality

This study utilised population-level data; thus, findings are generalisable to women in the CJS in England and Wales. The tool developed related to the presence of characteristics of personality difficulties, and these were based on women already accessing the OPD Pathway. As such, whilst data from the entire female CJS caseload were analysed, this is a predominantly White population and psychological complexities indicative of personality difficulty may differ across ethnic groups. The resultant items comprising the new identification criteria may, therefore, not be as applicable to other, more ethnically diverse populations than that found in England and Wales.

## Conclusion

Andrews et al. noted that criminal justice programmes that do not address the major criminogenic needs may cause increased reoffending whereas those informed by the Risk–Need–Responsivity principles have been found to reduce recidivism (53). Programmes tailored towards individuals struggling with personality difficulties must therefore consider both risk and protective factors in women presenting with complex psychological needs. Items identifying women for inclusion in the OPD Pathway programme directly implicate such factors as accommodation issues, lifestyle characteristics, and relationship difficulties, and yet a system that favours short custodial sentences over diversion programmes and community-based rehabilitation services could only aggravate these issues (54). Individuals presenting with the features identified in the new women’s OPD criteria, such as emotionally driven offending, excessive violence, perpetration of domestic violence, and arson, are clearly a highly complex cohort requiring dedicated psychological resources to help address underlying causes in an effort to reduce risks and improve wellbeing. Programmes such as the OPD Pathway must endeavour to work with women across custodial and community settings, ensuring support is maintained throughout transition periods and that those responsible for the management of high-risk women are themselves supported to maintain relational consistency (55). Tailoring programmes to adapt to these factors and implement transitional services to provide continued access to appropriate support upon release will be crucial to tackling prolific reoffending by such groups. Revising the OPD identification criteria for women has resulted in a tool that more appropriately aligns with current research on factors associated with serious offending by women. This move away from a process clearly aimed at men should contribute to the development and provision of better-designed rehabilitation services.

## Data Availability

The original contributions presented in the study are included in the article/supplementary material. Further inquiries can be directed to the corresponding author.

## Footnotes

1 Management of individuals subject to MAPPA involves police, probation, and prison services with additional cooperation from local education, housing authorities, and others. At the time of analysis, MAPPA were structured around three offence categories (sexual, violent, and other dangerous offences) and three levels of management requiring varying degrees of interagency responsibility.
